# Interaction between allelic variations in vitamin D receptor and retinoid X receptor genes on metabolic traits

**DOI:** 10.1186/1471-2156-15-37

**Published:** 2014-03-19

**Authors:** Karani S Vimaleswaran, Alana Cavadino, Diane J Berry, Massimo Mangino, Peter Andrews, Jason H Moore, Timothy D Spector, Chris Power, Marjo-Riitta Järvelin, Elina Hyppönen

**Affiliations:** 1 Centre for Paediatric Epidemiology and Biostatistics, UCL Institute of Child Health, London, UK; 2 Hugh Sinclair Unit of Human Nutrition, Department of Food & Nutritional Sciences, School of Chemistry, Food & Pharmacy, University of Reading, Whiteknights, PO Box 226, Reading RG6 6AP, UK; 3 Department of Twin Research and Genetic Epidemiology, King’s College London, London, UK; 4 Institute for Quantitative Biomedical Sciences, Department of Genetics, Geisel School of Medicine, Dartmouth College, Hanover, NH, 03756, USA; 5 Department of Epidemiology and Biostatistics, MRC Health Protection Agency Centre for Environment and Health, School of Public Health, Imperial College London, London, UK; 6 Institute of Health Sciences, University of Oulu, P.O. Box 5000, FI-90014 Oulu, Finland; 7 Biocenter Oulu, University of Oulu P.O. Box 5000, Aapistie 5A, FI-90014 Oulu, Finland; 8 Unit of Primary Care, Oulu University Hospital, Kajaanintie 50, P.O. Box 20 FI-90220 Oulu, Finland; 9 Department of Children, Young People and Families, National Institute for Health and Welfare, Box 310, 90101 Oulu, Finland; 10 School of Population Health and Sansom Institute, University of South Australia; South Australian Health and Medical Research Institute, Adelaide, Australia

**Keywords:** VDR, RXRG, SNPs, SNP-SNP interaction, 1958BC

## Abstract

**Background:**

Low vitamin D status has been shown to be a risk factor for several metabolic traits such as obesity, diabetes and cardiovascular disease. The biological actions of 1, 25-dihydroxyvitamin D, are mediated through the vitamin D receptor (VDR), which heterodimerizes with retinoid X receptor, gamma (RXRG). Hence, we examined the potential interactions between the tagging polymorphisms in the *VDR* (22 tag SNPs) and *RXRG* (23 tag SNPs) genes on metabolic outcomes such as body mass index, waist circumference, waist-hip ratio (WHR), high- and low-density lipoprotein (LDL) cholesterols, serum triglycerides, systolic and diastolic blood pressures and glycated haemoglobin in the 1958 British Birth Cohort (1958BC, up to n = 5,231). We used Multifactor- dimensionality reduction (MDR) program as a non-parametric test to examine for potential interactions between the *VDR* and *RXRG* gene polymorphisms in the 1958BC. We used the data from Northern Finland Birth Cohort 1966 (NFBC66, up to n = 5,316) and Twins UK (up to n = 3,943) to replicate our initial findings from 1958BC.

**Results:**

After Bonferroni correction, the joint-likelihood ratio test suggested interactions on serum triglycerides (4 SNP - SNP pairs), LDL cholesterol (2 SNP - SNP pairs) and WHR (1 SNP - SNP pair) in the 1958BC. MDR permutation model testing analysis showed one two-way and one three-way interaction to be statistically significant on serum triglycerides in the 1958BC. In meta-analysis of results from two replication cohorts (NFBC66 and Twins UK, total n = 8,183), none of the interactions remained after correction for multiple testing (P_interaction_ >0.17).

**Conclusions:**

Our results did not provide strong evidence for interactions between allelic variations in *VDR* and *RXRG* genes on metabolic outcomes; however, further replication studies on large samples are needed to confirm our findings.

## Background

Low vitamin D status has become a major public health problem due to its associations with several chronic diseases such as diabetes [1] and cardiovascular disease [2]. Observational studies have provided evidence for an association of serum 25-hydroxyvitamin D [25(OH)D] concentrations with blood pressures [3] and lipid outcomes [4]. These findings suggest a role of vitamin D in mediating biological functions required for the normal functioning of the body.

**Figure 1:**
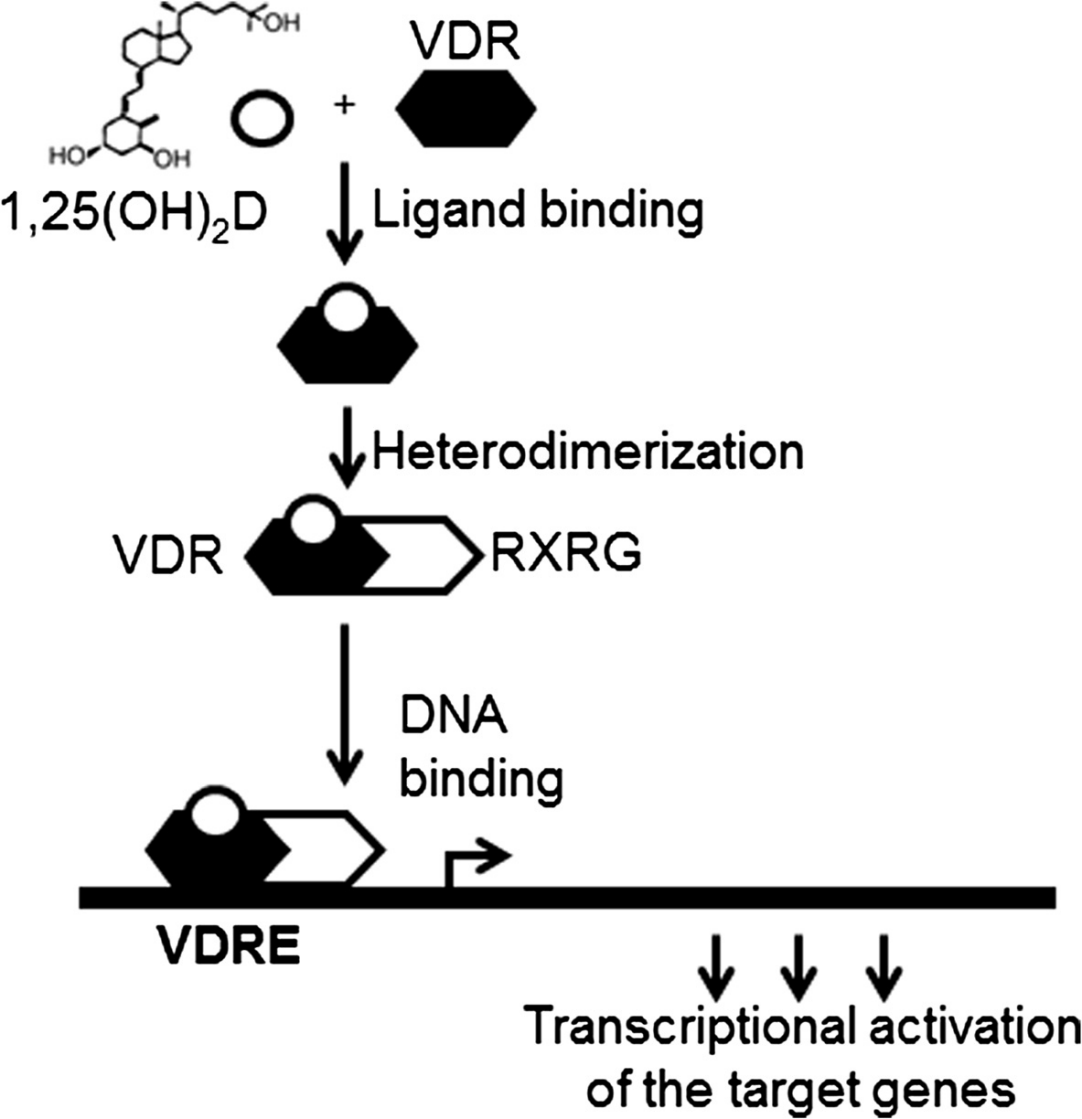
**Key stages involved in transcriptional regulation of 1,25**-**dihydroxyvitamin D (1,25(OH)**_**2**_**D).** The ligand (1,25(OH)_2_D) binds to the vitamin D receptor (VDR), which stimulates the heterodimerization of VDR with retinoid X receptor gamma (RXRG), followed by binding of the VDR/RXRG complex to the vitamin D response element (VDRE) and leading to the transcriptional activation of the target genes.

Vitamin D is involved in a variety of biological actions such as calcium metabolism, cell proliferation and differentiation [5]. Vitamin D, that is derived from the diet or by bio-activation of 7-dehydrocholesterol, must be activated to exert its biological activity [6]. The most active metabolite of vitamin D is calcitriol, 1,25-dihydroxyvitamin D (1,25(OH)_2_D), the genomic actions of which are mediated through the ligand-activated transcription factor, vitamin D receptor (VDR) [7]. 1,25(OH)_2_D mediates its action as a ligand by binding to the VDR, which regulates the transcription of the target genes by heterodimerizing with retinoid X receptor (RXR) (Figure 1). This VDR-RXR complex interacts with the hexameric DNA sequence element, vitamin D response elements (VDREs), which are found in the promoter regions of the target genes [8] (Figure 1). Thus, genetic alterations of *VDR* and *RXR* genes could lead to important defects in gene activation, cell proliferation and differentiation, calcium homeostasis and other related biological mechanisms.

Based on the biological relationship between VDR and RXR (Figure 1), we hypothesised that genetic variations in *VDR* and *RXR* genes have an effect on metabolic outcomes. In this paper, we examine the potential interactions between tagging polymorphisms in the *VDR* and *RXRG* genes on metabolic traits such as BMI, waist circumference (WC), waist hip ratio (WHR, adjusted for BMI), high- (HDL) and low- (LDL) density lipoprotein cholesterols, serum triglycerides, systolic (SBP) and diastolic (DBP) blood pressures and glycated haemoglobin (HbA1c).

## Methods

### Study population

We used information from the 1958 British birth cohort (1958BC, up to n = 5,231) as the discovery sample, and from the Northern Finland Birth cohort 1966 (NFBC66, up to n = 5,316) and Twins UK (up to n = 3,943)] to replicate the initial findings from the 1958BC.

#### 1958BC

Detailed description of the 1958 British birth cohort (1958BC) has been published previously [9]. In brief, study participants were born in England, Scotland or Wales during one week in March 1958 (n = 17,638). At age 45 years, 11,971 participants were invited to attend a biomedical survey: 9,377 (78%) completed at least one questionnaire. The 1958BC is almost entirely a white European population (98%) [10], and for these analyses, 158 individuals of other ethnic groups and one pregnant participant were excluded. The 45-year biomedical survey was approved by the South-East Multi-Centre Research Ethics Committee (ref. 01/1/44), the ethics approval for genetic work was granted by the Joint UCL/UCLH Committees on the Ethics of Human Research (Committee A) Ref: 08/H0714/40, and written consent [for use of information in medical research studies] was obtained from the participants. For the present study, all the analyses were performed in up to 5,231 individuals.

#### NFBC66

The Northern Finland Birth Cohort of 1966 (NFBC66) comprises a total of 12,058 live-births to mothers living in the two northern‒most provinces of Finland, who were invited to participate if they had expected delivery dates during 1966 [11]. At age 31 all individuals still living in Northern Finland or the Helsinki area were asked to participate in a detailed biological and medical examination (n = 6,007) as well as a questionnaire. The University of Oulu ethics committee approved the study. The present study includes up to 5,316 individuals with genotype data and information on WHR, serum triglycerides and LDL cholesterol. Written informed consent was obtained from all the participants and the Ethics Committee of the Faculty of Medicine at the University of Oulu approved the study.

#### Twins UK

The Twins registry in St. Thomas' Hospital, King's College London recruited a total sample of 11,000 identical and non-identical, mostly female Caucasian, twins from across the UK through national media campaigns [12]. Their age ranges between 16 and 85 years. Over 7,000 twins have attended detailed clinical examinations with a wide range of phenotypes over the last 18 years. All participants were recruited without presence or interest in any particular disease or trait. We included individuals for whom data on WHR (n = 3,943), serum triglycerides (n = 1,996) or LDL cholesterol (n = 1,992) were available. The Guy’s and St Thomas’ (GSTT) Ethics Committee approved the study and all the study participants gave informed consent.

### Measurements

#### 1958BC

Weight and standing height, at 45 years of age, were measured without shoes and in light clothing by a trained nurse using standardized protocols and equipment; waist circumference was measured by the nurse midway between the costal margin and iliac crest. BMI was calculated as weight (kg)/height (m)^2^. Blood pressure was measured in a seated position, after 5 min rest, using an Omron 705CP automated sphygmomanometer with a large cuff for participants with a mid-upper arm circumference ≥32 cm; the measurement was repeated three times, and blood pressure was determined as the average of successful measurements.

Venous blood samples were drawn without prior fasting and posted to the collaborating laboratory. Glycosylated haemoglobin (HbA1c) was assayed using high-performance liquid chromatography standardized to the Diabetes Control and Complications Trial [13]. Triglycerides and HDL cholesterols were measured by standard autoanalyzer methodology.

#### NFBC66

Height and body weight were measured using a standardized height measure and scale. The participants were asked to fast overnight before a blood sample was taken. Serum HDL cholesterol and triglycerides were determined by enzymatic methods using a Hitachi 911 Clinical Chemistry Analyzer (Boehringer Mannheim). Serum LDL was calculated by the Friedewald formula if the serum TG level was <354 mg/dl; if the triglyceride level was <354 mg/dl, LDL was determined by precipitating LD-lipoproteins with heparin and measuring cholesterol in the liquid phase and subtracting it from total cholesterol.

#### Twins UK

Weight and standing height were measured without shoes and in light clothing by a trained nurse. Blood sample collection for determination of fasting lipids was drawn from most subjects after a minimum 8-h overnight fast. Serum was stored at −45°C until analyzed using a Cobas Fara machine (Roche Diagnostics, Lewes, UK). A colourimetric enzymatic method was used to determine total cholesterol, triglycerides and HDL cholesterol levels. The latter was measured after precipitation from chylomicron, LDL and VLDL particles by magnesium and dextran sulphate.

### Tag SNP selection

Tag SNPs for *VDR* and *RXRG* genes were chosen using the genotype data from the International HapMap collected in individuals of Northern and Western European ancestry (CEU) (HapMap data release 24/ phase II Nov08, on NCBI B36 assembly, dbSNP b126). The Haploview software V3.3 ( http://www.broadinstitute.org/haploview/haploview-downloads) was used to assess the linkage disequilibrium (LD) structure between SNPs [14]. Tagger software was used to select tag SNPs with the ‘pairwise tagging only’ option and an r^2^ threshold of >0.8 (±10 kb upstream and downstream of the genes). In the tag SNP selection, we force included the functional SNPs (*VDR* SNPs: rs731236 and rs2228570; *RXRG* SNPs: rs2134095) previously studied [15-18] before running tagger. There were 30 *VDR* and 31 *RXRG* tag SNPs; however, after applying the quality control criteria [call rate >99% for genotyped SNPs, average genotype probability across all individuals in the sample >90% for imputed SNPs and minor allele frequency >5%], there were only 22 *VDR* and 23 *RXRG* tag SNPs.

### SNP genotyping

#### 1958BC

Genome-wide data for the 1958BC were obtained through two sub-studies, both using the 1958BC participants as population controls. The first sub-study included 3000 DNA samples randomly selected as part of the Welcome Trust Case Control Consortium (WTCCC2) and genotyped on the Affymetrix 6.0 platform [19]. The second sub-study was the Type 1 diabetes case–control study (T1DGC) which used 2,500 DNA samples and genotyped using the Illumina Infinium 550 K chip through the JDRF/WT Diabetes and Inflammation Laboratory (DIL) [20]. IMPUTE was used for the imputations that were done in the 1958BC.

#### NFBC66

For NFBC, genomic DNA was extracted from whole blood using standard methods. All DNA samples for the Illumina Infinium 370cnvDuo array were prepared for genotyping by the Broad Institute Biological Sample Repository (BSP). The 1000 Genome imputation was carried out for the NFBC66 samples using IMPUTE2.

#### Twins UK

Genotyping of the TwinsUK dataset was done with a combination of Illumina arrays (HumanHap300, HumanHap610Q, 1 M-Duo and 1.2 M Duo 1 M). The normalised intensity data for each of the three arrays were pooled separately (with 1 M-Duo and 1.2 M Duo 1 M pooled together). For each dataset, the Illluminus calling algorithm was used to assign genotypes in the pooled data. No calls were assigned if an individual's most likely genotype was called with less than a posterior probability threshold of 0.95. Prior to merging, pairwise comparison was performed among the three datasets. Further exclusion of SNPs and samples was done to avoid spurious genotyping effects, identified as follows: (i) concordance at duplicate samples <1% (i.e., only samples with ≥99% concordance included for the study); (ii) concordance at duplicate SNPs <1% (i.e., only SNPs with ≥99% concordance included for the analysis); (iii) visual inspection of QQ plots for logistic regression applied to all pairwise dataset comparisons; (iv) Hardy-Weinberg p-value <10^−6^, assessed in a set of unrelated samples; (v) observed pairwise IBD probabilities (samples excluded if the IBD threshold was less than 0.30) suggestive of sample identity errors.

### Statistical analysis

The natural logarithm was used to transform slightly skewed metabolic measures (BMI, WC, WHR, HbA1c and serum triglycerides) to approximate a normal distribution. All the SNPs were coded additively and with the effect allele as the minor allele. Linear regression models were used to evaluate the interaction between the *VDR* and *RXRG* tag SNPs on the following outcomes: BMI, WC, WHR, SBP, DBP, HDL and LDL cholesterol, serum triglycerides and HbA1c. The Friedewald equation was used to calculate LDL cholesterol levels in subjects with triglycerides ≤4.52 mmol/L [21]. In the 1958BC, linear regression models were adjusted for gender, geographical region (coded as Scotland, North, Middle, and South of England including Wales, and London) and genotyping platform. The use of medication was adjusted for in the models of HbA1c, serum triglycerides, LDL and HDL cholesterols. Serum triglyceride measures were further adjusted for time since eating prior to blood sample. Blood pressure of individuals who were on blood pressure medication was adjusted by adding 15 mm Hg to SBP and 10 mm Hg to the DBP [22]. Models for WHR were adjusted for BMI to test whether the effects of the SNP-SNP interactions on WHR are independent of BMI.

A joint likelihood ratio test (LRT) of the main SNP effects and the SNP-SNP interaction effects was used in the linear regression analyses to maximise statistical power (H0: βS1 = βS2 = βS1xS2 = 0) [23]. In comparison to the joint LRT of the main and the interaction effects, we also performed direct LRT tests for interaction (one degree of freedom test, H0: βS1xS2 = 0). This was done by comparing the model with the SNP-SNP interaction term and the marginal effects of both SNPs, with a model including the marginal effects of both SNPs only. Bonferroni correction was applied to p-values in order to account for multiple testing (22 × 23 = 506 SNP-SNP combinations assessed). Combinations with a corrected p-value <0.05 (uncorrected P < 0.05/506 = 9.9 × 10^−5^) were selected for replication.

At the discovery stage, we also used Multifactor Dimensionality Reduction (MDR) program (version 3.0.3) [24,25] as a non-parametric test to scan for potential interactions (one to four way combinations) between the *VDR* and *RXRG* tag SNPs on all the metabolic traits in the 1958BC. MDR program is a genetic model free approach [24,25], and includes a combined cross-validation and permutation testing procedure. With 10-fold cross-validation, the data are divided into 10 equal parts, and the model is developed on 9/10 of the data (training set) and then tested on 1/10 of the remaining data (testing set). The cross-validation consistency was done as a measure of how many times out of 10 divisions of the data MDR finds the same best model; hence, the higher the consistency, the better the model. Permutation testing was performed to assess the probability of obtaining a testing accuracy as large as or larger than that observed in the original data, given that the null hypothesis of no association is true. This is carried out by randomizing the samples 1000 times and repeating the MDR analysis on each randomized dataset. This process yields an empirical distribution of testing accuracies under the null hypothesis, which is in turn used to calculate a p-value. For the MDR analysis in the 1958BC (up to 5,231 individuals), trait values were standardised for covariates using the same adjustments as in linear regression analyses (see above), as MDR analysis does not take account of these covariates. This was done by regressing the relevant covariates on the trait and using the standardised residuals as the new “trait” outcome variables for use in the MDR analyses.

For replication analyses, a one degree of freedom test for the interaction term was used. In the NFBC66, models were adjusted for gender, population substructure (using the first two principal components) and the use of lipid lowering medications, as described above. Available covariates in Twins UK were gender and age. The cluster function for familial relatedness was used to account for non-independence of the twin pairs in analyses for Twins UK. Results from the two replication cohorts (NFBC66 and Twins UK) were then meta-analysed using the inverse-variance method for a fixed effects model. Bonferroni correction was also applied in order to account for multiple testing [P < 0.05/8 for 8 combinations assessed]. All analyses were carried out using STATA, version 12, except for the analyses in Twins UK, where STATA, version 10 was used.

The power to detect SNP-SNP interactions in the 1958BC (n =5,231) was calculated using the Quanto software (version 1.2.4). The power to detect SNP-SNP interactions for a standard normal outcome was calculated with different combinations of minor allele frequency (MAF) and an interaction beta of up to 0.25 (marginal SNP effects were set to 0.02). There was 80% power to detect an interaction β as small as 0.08 when both SNPs had a MAF of 0.40. However, when both SNPs had a MAF of 0.1, there was 80% power to detect interaction effect sizes only as small as 0.22 (Additional file 1: Figure S1). Despite the large sample size in the 1958BC, we lack the power to detect smaller interactions, particularly when looking for combinations of SNPs with lower MAF.

## Results

### Main effects

In the 1958BC (n = 5,231), after correction for multiple testing, only two *RXRG* SNPs (rs3753898 and rs283695) showed a significant association with WHR (adjusted for BMI) and LDL, respectively. None of the *VDR* or other SNPs from *RXRG* was associated with any of the metabolic traits (Additional file 2: Table S1, Additional file 3: Table S2).

### SNP-SNP interaction analysis in the discovery cohort (1958BC)

In the 1958BC, the joint LRT in linear regression analyses showed significant SNP-SNP interactions on serum triglycerides (4 SNP-SNP pairs), LDL cholesterol (2 SNP-SNP pairs) and WHR (1 SNP-SNP pair) after correction for multiple testing (Table 1). There were no significant interactions based on 1df interaction test in linear regression after correction for multiple testing (Additional file 4: Table S3).

**Table 1:** **Interaction analysis between vitamin D receptor ( *VDR *) and retinoid X receptor-gamma ( *RXRG *) polymorphisms in the discovery cohort (1958 British Birth Cohort) using linear regression**

**Outcome**	***VDR***	***RXRG***	**N in analysis**	**Interaction**	Unadjusted P (adjusted P*) from joint LRT
	**SNP**	**SNP**		**Beta ± SE**	
Low density lipoprotein cholesterol	rs3847987	rs283695	4,700	−0.12 ± 0.04	1.0x10^−5^ (0.03)
	rs11574143	rs283695	4,696	−0.13 ± 0.04	2.9x10^−5^ (0.04)
Serum triglycerides	rs11574143	rs17429123	4,843	0.13 ± 0.04	4.5x10^−5^ (0.01)
	rs11574143	rs10918172	4,873	0.13 ± 0.04	5.7x10^−5^ (0.02)
	rs3847987	rs17429123	4,849	0.12 ± 0.03	7.1x10^−5^ (0.02)
	rs3847987	rs10918172	4,880	0.12 ± 0.03	7.5x10^−5^ (0.05)
Waist Hip Ratio	rs2283342	rs157872	5,067	−0.01 ± 0.002	7.7x10^−5^ (0.04)

*Bonferroni correction applied to P values.

MDR program was used as an additional method (non-parametric approach) to test for the potential interactions (one-way to four-way) between *VDR* and *RXRG* genes on the metabolic traits in the 1958BC. Using this method, we identified one two-way (rs11574143, rs10918172) and one three-way (rs739837, rs2238136, rs12739596) interaction on serum triglycerides (Table 2). The two-way interaction (rs11574143, rs10918172) on serum triglycerides in the MDR analysis was consistent with the results from the joint LRT (joint LRT, uncorrected p-value = 5.7 × 10^−5^; corrected p-value: 0.02).

**Table 2:** **Interaction analysis between vitamin D receptor ( *VDR *) and retinoid X receptor-gamma ( *RXRG *) polymorphisms in the discovery cohort (1958 British Birth Cohort) using Multifactor Dimensionality Reduction (MDR) program**

**Outcome**	***VDR***	***RXRG***	**T-Statistic**	**Cross validation consistency**	**P value**
	**SNP**	**SNP**			
Serum triglycerides	rs11574143	rs10918172	4.72	9/10	0.035-0.036
	rs739837, rs2238136	rs12739596	6.32	9/10	0.033-0.034

### Replication of the SNP-SNP interaction findings in the NFBC66 and Twins UK

In replication analyses using NFBC66 and Twins UK (total n = 8,183), none of the interactions identified from the joint LRT were significant after correction for multiple testing (Table 3). Without correction, the interaction between the *VDR* SNP rs3847987 and the *RXRG* SNP rs10918172 on serum triglycerides was borderline (meta-analysis 1df test for interaction, p-value = 0.02, Table 3).

**Table 3:** **Interaction between vitamin D receptor ( *VDR *) and retinoid X receptor-gamma ( *RXRG *) polymorphisms in the replication cohorts (Northern Finland Birth Cohort 1966 and Twins UK)**

**Outcome**	***VDR***	***RXRG***	**Northern Finland Birth Cohort 1966 (NFBC66)**			**Twins UK**			**Meta-analysis of the results from NFBC66 and Twins UK**	
	**SNP**	**SNP**								
			**N in analysis**	**Interaction Beta ± SE**	**P**_**interaction**_	**N in analysis**	**Interaction Beta ± SE**	**P**_**interaction**_	**Interaction Beta ± SE**	**Unadjusted P**_**interaction **_**(Adjusted P**_interaction_*)
Low density lipoprotein cholesterol	rs3847987	rs283695	5,007	−0.03 ± 0.03	0.26	1,555	−0.08 ± 0.08	0.31	−0.04 ± 0.03	0.16
	rs11574143	rs283695	4,990	−0.02 ± 0.03	0.52	1,528	−0.03 ± 0.08	0.75	−0.02 ± 0.03	0.48
Serum triglycerides	rs11574143	rs17429123	5,155	0.01 ± 0.03	0.74	1,686	0.09 ± 0.06	0.13	0.03 ± 0.03	0.32
	rs11574143	rs10918172	5,131	0.03 ± 0.03	0.28	1,725	0.05 ± 0.05	0.37	0.04 ± 0.03	0.16
	rs3847987	rs17429123	5,172	0.03 ± 0.03	0.28	1,730	0.08 ± 0.05	0.09	0.05 ± 0.02	0.08
	rs3847987	rs10918172	5,148	0.05 ± 0.03	0.07	1,767	0.07 ± 0.05	0.14	0.06 ± 0.03	0.02 (0.17)
	rs739837, rs2238136	rs1273959	5,204	−0.002 ± 0.03	0.95	1,883	0.01 ± 0.04	0.79	0.003 ± 0.03	0.91
Waist hip ratio	rs2283342	rs157872	4,707	0.002 ± 0.003	0.53	3,376	−0.002 ± 0.004	0.62	0.001 ± 0.002	0.78

*Bonferroni correction applied to P_interaction_ values.

In replication analyses using NFBC66 and Twins UK, none of the interactions identified from the MDR analysis were significant (meta-analysed P_interaction_ > 0.16, for all comparisons) (Table 3).

## Discussion

To our knowledge, this is the first study to examine interactions between *VDR* and *RXRG* tag SNPs on metabolic traits. VDR is the key nuclear receptor for the active hormonal form of vitamin D [1,25-dihydroxyvitamin D_3,_ 1,25(OH)_2_D_3_] and there is some previous evidence for its role in obesity- and diabetes- related traits [26,27]. However, despite the known contribution of vitamin D on bone metabolism and VDR being the key hormonal receptor [5], previous studies have failed to identify an association between *VDR* polymorphisms and bone-related outcomes such as bone mineral density (BMD) [28,29]. One of the reasons for the lack of association between *VDR* and BMD could be due to a compensatory interaction with *RXRG* polymorphism, which was also the rational for undertaking analyses presented in the current study.

RXR functions as a master regulator of various signalling pathways through heterodimerization with VDR. Various isoforms of the RXR serve as dimeric partners for VDR binding to VDREs [30]; however it has been shown that VDR binds to RXRG more avidly than other RXR isoforms [30]. Animal studies have shown that VDR(−/−)/RXRG(−/−) mice exhibit features typical of vitamin D-dependent rickets type II, including growth retardation, impaired bone formation, hypocalcemia, and alopecia [31]. Compared to VDR(−/−) mice, growth plate development in VDR(−/−)/RXRG(−/−) mutant mice was more severely impaired [31]. In addition, genome-wide association scans have identified *RXRG* variants to be associated with various human phenotypic traits such as age at menarche [32], rapid disease progression in patients with human immunodeficiency virus type 1 infection [33] and personality trait [34], suggesting a role of RXRG in mediating multiple signalling pathways. Also, meta-analysis of inter-species liver co-expression networks identified a human-specific sub-network regulated by RXRG, which has been validated to play a role in hyperlipidemia and type 2 diabetes [35]. Hence, based on this biological evidence and the role of VDR and RXRG as heterodimer receptor partners, it was anticipated that defects in *VDR* or *RXRG* genes might have an effect on the biological interaction which, in turn, could affect metabolic pathways implicated in obesity, diabetes or cardiovascular traits.

We observed several possibly interesting SNP-SNP interactions in the 1958BC using linear regression analysis on various metabolic outcomes such as WHR, serum triglycerides and LDL cholesterol. Furthermore, one additional three-way interaction was identified in the 1958BC using MDR permutation model testing analysis, a computational approach to detect and characterize interactions [24]. In the replication meta-analysis of the two cohorts (NFBC66 and TwinsUK), none of the interactions remained significant except for one SNP-SNP two-way borderline interaction on serum triglycerides (*VDR* rs3847987 – *RXRG* rs10918172, P_interaction_ = 0.02). However, this two-way interaction was not significant after correction for multiple testing (P_interaction_ = 0.17). Although our finding of the two-way interaction on serum triglycerides (before correction for multiple testing) and the association of *RXRG* SNP rs283695 with LDL cholesterol (main effect) supports the hypothesis that *VDR* and *RXRG* are strong candidates for lipid metabolism [35-37], these results should be dealt with caution and need further confirmation using large samples.

An important challenge with SNP-SNP interaction arises from the large number of statistical tests involved, thereby leading to the requirement of significant thresholds to control for type 1 error. Hence, only substantial joint effects are likely to be detected, unless sample sizes are very large. In the present study, despite using large samples in both discovery (1958BC, n = 5,231) and replication analyses (NFBC66 + Twins UK, total n up to 8,183), none of the interactions remained significant after correction for multiple testing. Given that vitamin D has been shown to be associated with various metabolic outcomes, we also re-ran the joint LRT analyses in the 1958BC adjusting for 25(OH)D concentrations and found that our results remain unchanged (data not shown). Overall, the participants were relatively young, with some heterogeneity across the three studies, and it is possible that stronger effects could be seen in older populations when the metabolic risks associated with ageing are more firmly established.

## Conclusions

Our results do not provide strong evidence for interactions between the allelic variations in the *VDR* and *RXRG* genes on metabolic traits; however, further replication studies are highly warranted on large samples to confirm our findings.

## Abbreviations

VDR: Vitamin D receptor; RXRG: Retinoid X receptor, gamma; 1958BC: 1958 British Birth Cohort; SNP: Single nucleotide polymorphism; BMI: Body mass index; LDL: Low density lipoprotein; HDL: High density lipoprotein; HbA1c: Glycated haemoglobin; WHR: Waist-hip ratio MDR, Multiple Dimensionality Reduction

## Competing interests

The authors declare that they have no competing interests.

## Authors’ contributions

KSV and EH conceived and designed the study and drafted the manuscript; AC, DJB, JHM and PA provided input to the study design; AC, DJB, MM and PA performed the statistical analysis; JHM, TDS, CP, M-RJ provided critical review of the manuscript. All authors read and approved the final manuscript.

## Supplementary Material

Additional file 1: Figure S1 Calculation of the power to detect SNP-SNP interactions in the 1958 British Birth Cohort for a standard normal outcome with different combinations of minor allele frequency and an interaction beta of up to 0.25. Click here for file

Additional file 2: Table S1 a. Association between the Vitamin D Receptor polymorphisms and metabolic traits in the 1958 British Birth Cohort; b. Association between the Vitamin D Receptor polymorphisms [that showed a significant association (uncorrected for multiple testing) in the 1958BC] and metabolic traits in the NFBC66 and Twins UK. Click here for file

Additional file 3: Table S2 a: Association between the Retinoid X Receptor- gamma polymorphisms and metabolic traits in the 1958 British Birth Cohort; b: Association between the Retinoid X Receptor- gamma polymorphisms [that showed a significant association (uncorrected for multiple testing) in the 1958BC] and metabolic traits in the NFBC66 and Twins UK. Click here for file

Additional file 4: Table S3 SNP-SNP interactions in the 1958 British Birth Cohort based on one degree of freedom interaction test. Click here for file
