# Supplementary figures and images for: Interaction between allelic variations in vitamin D receptor and retinoid X receptor genes on metabolic traits
Source: BMC Genet. 2014 Mar 19;15:37. doi: 10.1186/1471-2156-15-37 (PMC4004151; doi:10.1186/1471-2156-15-37)

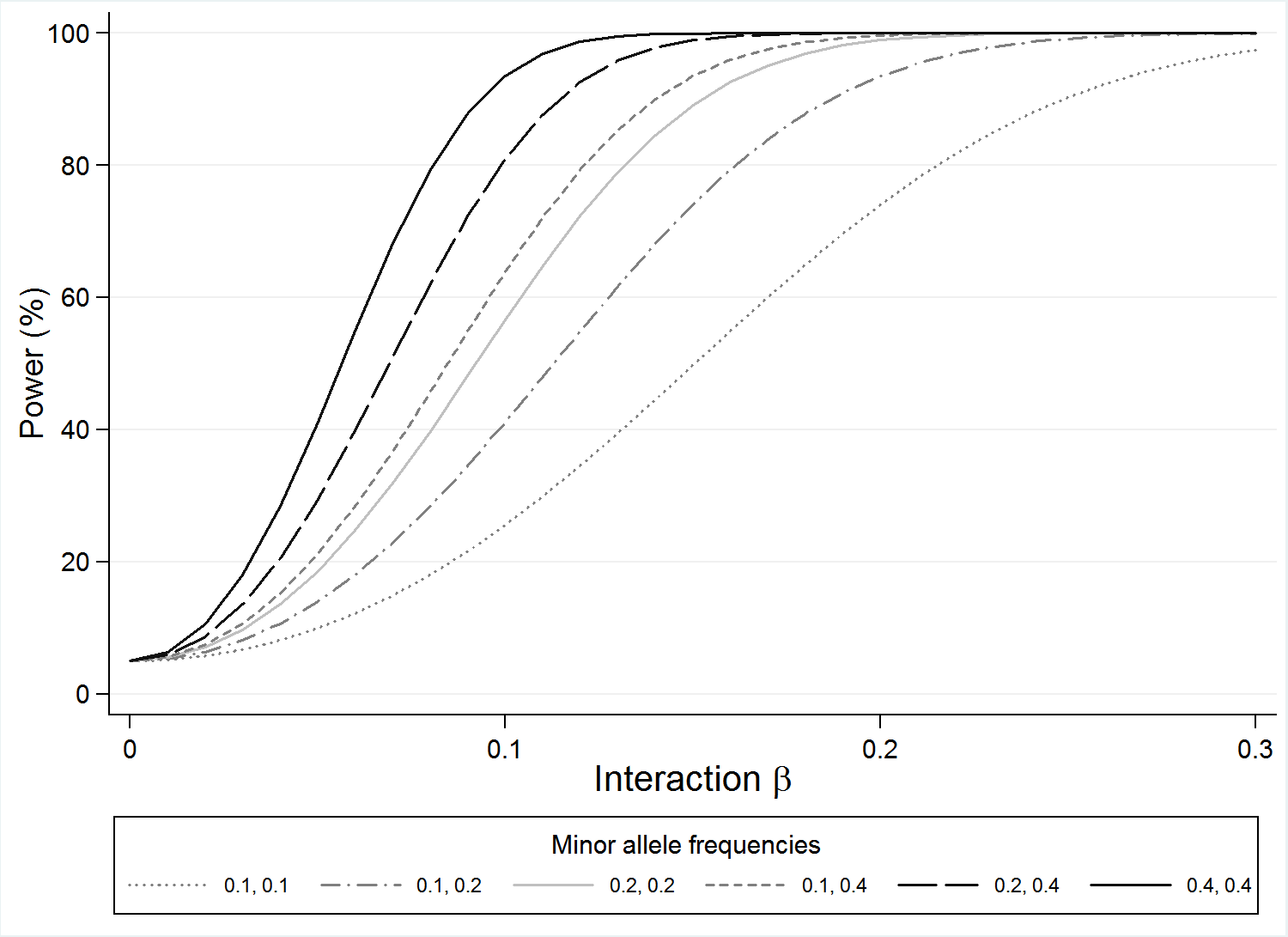

Supplement: Additional file 1: Figure S1 — Calculation of the power to detect SNP-SNP interactions in the 1958 British Birth Cohort for a standard normal outcome with different combinations of minor allele frequency and an interaction beta of up to 0.25. [file 1471-2156-15-37-S1.tiff]
